# Intestinal helminth infections in HIV-infected patients in Savannakhet after establishment of an HIV registration network in Lao People’s Democratic Republic

**DOI:** 10.1186/s41182-019-0142-0

**Published:** 2019-02-11

**Authors:** Yukako Kaneshiro, Khamphang Sourinphoumy, Naoki Imaizumi, Mangkhalar Rasaphon, Megumi Kuba-Miyara, Shugo Sakihama, Carmina Louise Hugo Guerrero, Ketsaphone Nhativong, Daisuke Nonaka, Tiengkham Pongvongsa, Jun Kobayashi, Sengchanh Kounnavong, Takuya Fukushima

**Affiliations:** 1 Laboratory of Hematoimmunology, Graduate School of Health Sciences, University of the Ryukyus, Nishihara, Okinawa Japan; 2 Savannakhet Provincial Hospital, Savannakhet, Lao People’s Democratic Republic; 3 Laboratory of Molecular Genetics, Graduate School of Health Sciences, University of the Ryukyus, Nishihara, Okinawa Japan; 4 Department of Pathology and Cell Biology, Graduate School of Medicine, Faculty of Medicine, University of the Ryukyus, Nishihara, Okinawa Japan; 5 Savannakhet Provincial Health Department, Savannakhet, Lao People’s Democratic Republic; 6 Department of Global Health, Graduate School of Health Sciences, University of the Ryukyus, Nishihara, Okinawa Japan; 7 National Institute of Public Health, Vientiane, Lao People’s Democratic Republic

**Keywords:** Human immunodeficiency virus, Helminth infections, Human T cell leukemia virus type I, Registration network, Lao People’s Democratic Republic

## Abstract

**Background:**

In Lao People’s Democratic Republic (PDR), which borders China, Vietnam, Cambodia, Thailand, and Myanmar, the number of HIV-infected patients has increased in recent years. HIV-infected patients diagnosed in Lao PDR are enrolled in a registration network and receive antiretroviral therapy (ART) covered by governmental financial support. Based on the registration network, we investigated intestinal helminth infections and coinfection with HTLV-1 in HIV-infected patients treated with an early intervention using ART in Lao PDR.

**Methods:**

This cross-sectional study of all 252 HIV-infected patients at Savannakhet Provincial Hospital, located in the southern part of Lao PDR, was conducted between February and March 2018. Socioepidemiological information and clinical information were collected from a registration network database and by questionnaire administered to participants. Microscopic examination of intestinal helminth infections in stool samples and particle agglutination for anti-HTLV-1 antibody in plasma were performed.

**Results:**

The median age of all 252 participants was 39 years old (range, 18–59). Based on the registration network database, there were 156 (61.9%) HIV-infected patients with a CD4-positive cell count ≥ 200 cells/μL and 146 (57.9%) with an HIV viral load < 250 copies/mL. Among 212 stool samples, 75 (35.4%) were found to contain one or more intestinal helminth species, including *Opisthorchis viverrini* (16.5%), *Strongyloides stercoralis* (10.8%), hookworm (10.4%), and *Taenia saginata* (3.3%). This rate of intestinal helminth infections was lower than that of a previous report conducted before the establishment of the registration network for HIV-infected patients in Lao PDR. There was no significant association between intestinal helminth infections and a lower CD4-positive T cell count or higher HIV viral load. HIV-infected patients with anti-HTLV-1 antibody positivity were not found in this cohort.

**Conclusion:**

The registration network and an early intervention using ART may provide good medical care and improve the clinical course of HIV-infected patients in Lao PDR. However, the incidence of intestinal helminth infections remains high at 35.4%. The development of a specific medical care system for helminth infection for HIV-infected patients is necessary.

## Introduction

**Fig. 1:**
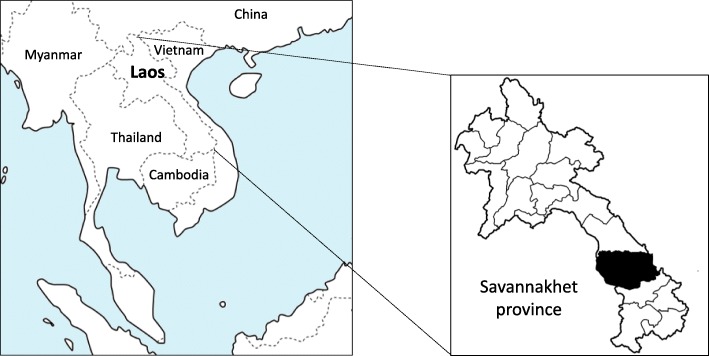
Location of Savannakhet Province (black) in Laos

Lao People’s Democratic Republic (Lao PDR) is a landlocked country in Southeast Asia that borders China, Vietnam, Cambodia, Thailand, and Myanmar (Fig. 1). Laos is currently a developing country and a geographical location heavily influenced by human transit and migration, which together have contributed to increase the rate of HIV infection [1]. In 2016, the prevalence of HIV carriers was estimated to be 0.3%, with the absolute number of people living with HIV/AIDS increasing every year [1, 2]. To grasp the current situation and to effectively treat HIV carriers and AIDS patients, a registration network covering the country has been implemented. Registered HIV carriers in this network receive antiretroviral therapy (ART) covered by governmental financial support, and most are followed up using parameters such as WHO stage, immune status (CD4-positive T cell count or HIV viral load), and type of ART [1].

HIV/AIDS is complicated by severe viral, fungal, bacterial, and parasitic infections. Intestinal parasitic infections are a serious public health problem in developing countries and an important cause of morbidity and mortality, particularly with the emergence of immunosuppressive diseases [3, 4]. Intestinal parasites are opportunistic complications and can cause severe episodes of diarrhea in patients with HIV/AIDS [5, 6]. Among the possible intestinal parasites, helminths are a public health concern in developing countries, producing a burden of disease that exceeds that of better-known conditions, including malaria and tuberculosis [7, 8]. Helminths have potent effects on the immune system and may have an important influence on other infections [9]. In addition, helminth infections show significant overlap with the geographical distribution of HIV [9]. However, the numbers of chronically infected individuals with helminths are often underestimated. In a recent survey before the development of the database for HIV-infected patients in Lao PDR, newly diagnosed HIV carriers without any treatment were at advanced stages and had a high incidence of intestinal parasites [10]. Some studies have proposed that T cell immunity involving CD4-positive T cells plays a role in defense against *Strongyloides stercoralis* [11, 12]. Hence, we hypothesized that the use of ART in HIV-infected patients protects against helminths such as *S. stercoralis* and that immune restoration after ART reduces the incidence and severity of parasitic diseases [13]. However, further follow-up was required to study the effect of ART on intestinal parasites in registered HIV carriers in Lao PDR.

Human T cell leukemia virus type I (HTLV-1) is a member of the human retrovirus family that includes HIV. It causes HTLV-1-associated diseases including adult T cell leukemia and HTLV-1-associated myelopathy. In Brazil, where HTLV-1 is endemic, the coinfection rate of HIV and HTLV-1/2 in 301,470 first-time blood donors was 2.4% [14]. Another report from Brazil demonstrated that, among 123 HIV-infected patients, 20 men (20.6%) and 6 women (23.1%) had detectable antibodies against HTLV-1/2 and that coinfection with HTLV-1/2 was associated with an increased risk of strongyloidiasis in HIV patients [15]. However, in Asian countries such as Lao PDR, there are limited data on the prevalence status of HTLV-1, much less the coinfection rate. In Indonesia, the coinfection rate of HIV and HTLV-1/2 was 1.3% (*n* = 5) in 375 drug abuser inmates in central Javan prisons [16].

Savannakhet Province is located in the southern part of Lao PDR and borders Vietnam to the east and Thailand to the west. Its capital, Nakhon Kaysone Phomvihane, forms an important trading post between Vietnam and Thailand (Fig. 1). Savannakhet Provincial Hospital, one of the seven HIV care and treatment centers in Lao PDR, treats HIV-infected patients with ART. Based on the registration network of HIV-infected patients in Lao PDR, we investigated intestinal helminth infections and the prevalence of HTLV-1 among registered HIV-infected patients receiving ART at Savannakhet Provincial Hospital.

## Methods

### Study sites and population

This cross-sectional study was carried out from February to March 2018 at Savannakhet Provincial Hospital in Lao PDR. The study population consisted of HIV carriers who were registered in the registration network in Lao PDR and aged over 18 years old for undergoing ART. Those with any severe condition or onset of AIDS were excluded from the study.

### Data collection

Individuals who agreed to participate in the study signed an informed consent form and answered a socioepidemiological questionnaire containing questions related to the following variables: age, sex, ethnic group, resident state, occupation, educational status, marital status, and infection route of HIV. Data on the CD4-positive T cell count, HIV viral load, and history of tuberculosis were obtained from the medical records of Savannakhet Provincial Hospital. For the data on the CD4-positive T cell count and HIV viral load, we adopted the latest measurement obtained within 1 year.

### Stool sample collection and analysis

Fresh stool samples were collected from each HIV-infected participant in clean dry universal bottles and transported as quickly as possible to the Savannakhet Provincial Hospital laboratory. The technicians of the hospital laboratories performed the direct smearing method in saline and immediately examined the samples under the microscope. Two experts evaluated one slide per patient; any slides judged by both experts to contain helminth larvae were considered to be helminth positive.

### Anti-HTLV-1 antibody analysis

Peripheral blood from all participants was collected, and serological diagnosis of HTLV-1 infection was performed using an anti-HTLV-1 antibody test kit, the SERODIA®-HTLV-I Particle Agglutination Kit (Fujirebio Inc., Tokyo, Japan).

### Statistical analysis

Analyses were performed using Statistical Package for the Social Sciences (version 20; SPSS Inc., Chicago, IL). Descriptive statistics were calculated. Frequencies were calculated for categorical variables. Proportions were compared using Fisher’s exact test. Differences were considered significant at *P* < 0.05.

### Ethics statement

**Fig. 2:**
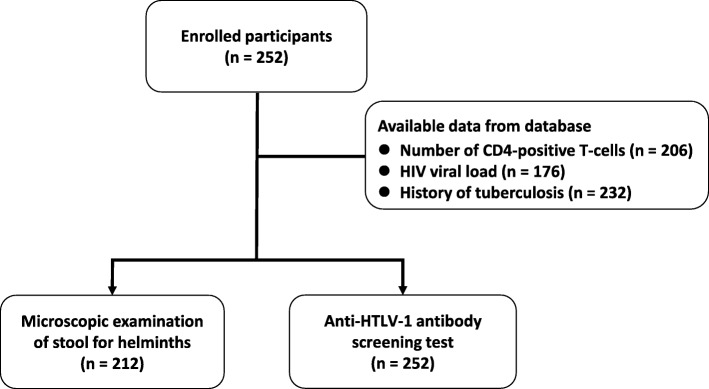
Workflow of this study

The study protocol (Fig. 2), including the consent procedure, was reviewed and approved by the Ethics Committee of the University of the Ryukyus (no. 169) and National Institute of Public Health Lao PDR (no. 2017. 88. MC). This research followed the principles expressed in the Declaration of Helsinki.

## Results

### Patient characteristics

A total of 252 HIV carriers were enrolled in this study and gave their consent: 138 (54.8%) were men and 114 (45.2%) were women. The median age was 39 years (range, 18–59 years). The demographic characteristics of the participants are summarized in Table 1. With respect to ethnicity, almost all participants were Lao Loum (94.8%). According to residential status, permanent residents and Laotian migrant workers living in Thailand comprised 181 (71.8%) and 71 (28.2%), respectively. The occupations of the participants were predominantly farming (51.2%) and merchant and company work (29.4%). Almost all participants had achieved either a primary (41.7%) or secondary (47.2%) education level. Almost all the HIV infection routes were sexually transmitted infections (94.8%). All participants were receiving ART, and 61.9% of participants (156 of 252) had a CD4-positive T cell count ≥ 200 cells/μL. Fifty-seven percent (146/252) of participants had an undetectable HIV viral load (< 250 copies/mL). All HIV carriers in Savannakhet Provincial Hospital were screened for tuberculosis using chest X-ray and underwent a tuberculin test after diagnosis of HIV. Of the 252 participants, 89 (38.4%) had a history of tuberculosis.

**Table 1:** Demographic and general characteristics of the study population (*n* = 252)

Sex (male/female)	138/114
Median age (range), years	39 (18–59)
Ethnic group	
Lao Loum	239 (94.8%)
Lao Theung	5 (2.0%)
Phou Thai	2 (0.8%)
Refused to answer	6 (2.4%)
Resident state	
Permanent resident	181 (71.8%)
Migrant*	71 (28.2%)
Occupation	
Farmer	129 (51.2%)
Merchant and company worker	74 (29.4%)
Government staff	12 (4.7%)
Student	7 (2.8%)
Unemployed	9 (3.6%)
Refused to answer	21 (8.3%)
Educational status	
None	13 (5.2%)
Primary	105 (41.7%)
Secondary	119 (47.2%)
College	1 (0.4%)
Refused to answer	14 (5.5%)
Marital status	
Single	55 (21.8%)
Married or living with partner	127 (50.4%)
Widowed or separated	64 (25.4%)
Refused to answer	6 (2.4%)
Infection route	
Sexual intercourse	239 (94.8%)
Mother-to-child transmission	5 (2.0%)
Unknown	8 (3.2%)
Number of CD4-positive T cells (cells/μL)	
< 200	43 (17.1%)
≥ 200	156 (61.9%)
No data	53 (21.0%)
HIV viral load (copies/mL)	
< 250	146 (57.9%)
≥ 250	12 (4.8%)
No data	94 (37.3%)
History of tuberculosis	
Positive	89 (38.4%)
Negative	143 (61.6%)

*Migrant worker: Laotian living and working in Thailand but receiving medical care in Savannakhet Province, Lao PDR

### Coinfection by HTLV-1

We screened for the anti-HTLV-1 antibody in all 232 participants, but HTLV-1 seropositivity was not detected.

### Intestinal helminth infection

Among all 252 participants, 212 stool samples were obtained and analyzed for the presence of intestinal helminths. Seventy-five patients (35.4%) were infected with one or more intestinal helminth species (Table 2). At 66.7% (12 of 18), the infection rate was higher in the 50–59 age group than in the other age groups: 40% (2/5) in the 18–19 group, 28.6% (10 of 35) in the 20–29 age group, 25.7% (18 of 70) in the 30–39 age group, and 39.3% (33 of 84) in the 40–49 age group. According to the occupation group, the infection rates of 37.3% (41 of 110) in farmers and 35.6% (21 of 59) in the merchant and company worker group were higher than the infection rates of 28.6% (2 of 7) in the student group, 25.0% (2 of 8) in the unemployed group, and 18.2% (2 of 11) in the government staff group. Among 74 helminth-infected patients with available data on fecal properties, 49 and 25 had loose or liquid and solid feces, respectively. Among the 212 HIV-infected patients who provided stool samples, *Opisthorchis viverrini*, found in 35 patients (16.5%), was the most common helminths, followed by *S. stercoralis* (23, 10.8%), hookworm (22, 10.4%), and *Taenia saginata* (7, 3.3%). Twelve (5.7%) showed double intestinal helminth infection (Table 3).

**Table 2:** Prevalence of intestinal helminths according to age, sex, and occupation in HIV-infected patients (*n* = 212)

	*n*	Helminth infection	
		Infected (%)	Uninfected (%)
		*n* = 75 (35.4%)	*n* = 137 (64.6%)
Sex			
Male	116	38 (32.8%)	78 (67.2%)
Female	96	37 (38.5%)	59 (61.5%)
Age, years			
18–19	5	2 (40.0%)	3 (60.0%)
20–29	35	10 (28.6%)	25 (71.4%)
30–39	70	18 (25.7%)	52 (74.3%)
40–49	84	33 (39.3%)	51 (60.7%)
50–59	18	12 (66.7%)	6 (33.3%)
Occupation			
Farmer	110	41 (37.3%)	69 (62.7%)
Marchant and company worker	59	21 (35.6%)	38 (64.4%)
Government staff	11	2 (18.2%)	9 (81.8%)
Students	7	2 (28.6%)	5 (71.4%)
Unemployed	8	2 (25.0%)	6 (75.0%)
Refused to answer	17	7 (41.2%)	10 (58.8%)

**Table 3:** The overall results of detected helminth infections among 212 HIV-infected patients

Species	Number of patients
Single infection	
*O. viverrini*	24 (11.3%)
*S. stercoralis*	18 (8.5%)
Hookworm	17 (8.0%)
*T. saginata*	4 (1.9%)
Double infection	
*O. viverrini* + *S. stercoralis*	4 (1.9%)
*O. viverrini* + hookworm	4 (1.9%)
*O. viverrini* + *T. saginata*	3 (1.4%)
*S. stercoralis* + hookworm	1 (0.5%)

### Association of intestinal helminths with the CD4-positive T cell count and the HIV viral load

We compared the intestinal helminth infection between individuals with a CD4-positive T cell count < 200 cells/μL and ≥ 200 cells/μL and between individuals with an HIV viral load count < 250 copies/mL and ≥ 250 copies/mL. However, neither a lower CD4-positive T cell count (Table 4) nor a higher HIV viral load (Table 5) was significantly associated with intestinal helminth infection.

**Table 4:** Association of intestinal helminths with the CD4-positive T cell count in HIV-infected patients (*n* = 199; among the participants who were screened for helminths via stool samples, only 199 had available CD4-positive T cell count data)

Species	CD4-positive T cell count (cells/μL)				OR (95% CI)	*P* value
	< 200 (*n* = 43)		≥ 200 (*n* = 156)			
	Positive	Negative	Positive	Negative		
Any helminths	14 (32.6%)	29 (67.4%)	56 (35.9%)	100 (64.1%)	0.86 (0.42–1.77)	0.72
*O. viverrini*	5 (11.6%)	38 (88.4%)	28 (17.9%)	128 (82.1%)	0.60 (0.22–1.67)	0.49
*S. stercoralis*	4 (9.3%)	39 (90.7%)	16 (10.3%)	140 (89.7%)	0.90 (0.28–2.84)	1.00
Hookworm	6 (14.0%)	37 (86.0%)	16 (10.3%)	140 (89.7%)	1.42 (0.52–3.88)	0.58
*T. saginata*	1 (2.3%)	42 (97.7%)	6 (3.8%)	150 (96.2%)	0.60 (0.07–5.08)	1.00

**Table 5:** Association of intestinal helminths with HIV viral load in HIV-infected patients (*n* = 158; among the participants who were screened for helminths via stool samples, only 158 had available HIV viral load data)

Species	HIV viral load (copies)/mL				OR (95% CI)	*P* value
	< 250 (*n* = 146)		≥ 250 (*n* = 12)			
	Positive	Negative	Positive	Negative		
Any helminth	52 (35.6%)	94 (64.4%)	3 (25.0%)	9 (75%)	0.60 (0.16–2.32)	0.54
*O. viverrini*	25 (17.1%)	121 (82.9%)	0 (0.0%)	12 (100%)	n.a.	0.22
*S. stercoralis*	14 (9.6%)	132 (90.4%)	1 (8.3%)	11 (91.7%)	0.86 (0.10–7.14)	1.00
Hookworm	17 (11.6%)	129 (88.4%)	2 (16.7%)	10 (83.3%)	1.52 (0.31–7.52)	0.64
*T. saginata*	5 (3.4%)	141 (96.6%)	0 (0.0%)	12 (100%)	n.a.	1.00

*n.a.* not applicable

## Discussion

This is the first screening study of intestinal helminth infections in HIV-infected patients treated with ART after the establishment of the HIV registration network in Lao PDR. Among 212 patients who provided stool samples, from a total of 252 participants, 75 (35.4%) were detected to have intestinal helminth infections, including 12 with double infections (5.7%).

The prevalence rate of intestinal helminth infection in this study (35.4%) was lower than that of a previous study (58.4%) in Lao PDR, which examined newly diagnosed HIV-infected patients before the development of the registration network and early intervention using ART [10]. In that report, more patients (83.9%) were in a progressive stage (3 or 4) according to the WHO clinical staging criteria of HIV [10], compared with almost all patients classified as stage 1 or 2 in this study. Furthermore, the median CD4-positive T cell count in the previous study (41 cells/μL) was lower than that in this study (357 cells/μL in 156 patients) [10]. These results suggested that early intervention using ART and systemic support for HIV-infected patients in Lao PDR could prevent the development of an immunocompromised status and reduce complications such as intestinal helminth infections. However, we may have underestimated the rate of helminth infection by neglecting to examine the presence of helminth eggs. To evaluate the rate more exactly, further study with the addition of the Kato-Katz technique to detect helminth eggs is needed.

In this study of HIV-infected patients, with few in an advanced stage, no significant association was found of CD4-positive T cell counts (< 200 cells/μL vs ≥ 200 cells/μL) and HIV viral load (< 250 copies/mL vs ≥ 250 copies/mL) with intestinal helminth infections. The previous cross-sectional study of helminth infections of 574 members of selected households in Saravane district, southern Laos, revealed a high prevalence of helminth infections: 88.7% of *O. viverrini*, 86.6% of hookworm, 32.9% of *Trichuris trichiura*, 9.8% of *Ascaris lumbricoides*, and 11.5% of *T. saginata*. The common types of parasite in this study—*O. viverrini*, hookworm, and *T. saginata*—were mostly the same as those of the previous report [17]. Because the baseline incidence of helminth infections in the citizens of this area remained high, early intervention using ART to control the HIV disease status might be able to only provide a minimal improvement in helminth infections. To clarify the population-based incidence of helminth infection in Savannakhet Province, a screening survey of citizens is warranted.

Otherwise, *S. stercoralis*, which was not a common helminth infection in the previous report of southern Laos [17], showed the second highest incidence of helminth infection in this study. Coinfection with *S. stercoralis* and HIV/AIDS has been reported in epidemiological studies [18–20]. Furthermore, severe infections known as hyperinfection with *S. stercoralis* affect immunocompromised people. On the other hand, Brown et al. [21] reported that treatment of immune reconstitution inflammatory syndrome (IRIS) after initiation of ART is associated with hyperinfection. These findings suggest that careful observation of the effectiveness of the early intervention using ART in HIV-infected patients with *S. stercoralis* is needed in Lao PDR.

This was the first screening survey of anti-HTLV-1 antibody in Lao PDR, and we could not find any HTLV-1 carrier among the 252 HIV-infected patients in Savannakhet Provincial Hospital. A similar survey of anti-HTLV-1 antibody in HIV-infected patients in Thailand also failed to find any HTLV-1 carriers [22, 23]. Our results indicate that Savannakhet Province, which borders Thailand, would not be an endemic area for HTLV-1. To confirm this finding, a further screening survey of anti-HTLV-1 antibody in donated blood is required.

## Conclusions

We conducted a detailed examination based on an HIV registration network, with the results suggesting that an HIV registration network and early intervention using ART might provide good medical care and improve the clinical course of HIV-infected patients in Lao PDR. However, the incidence of intestinal helminth infections remains high at 35.4%. Of the 252 participants with HIV, 28% were Laotian migrant workers living in Thailand, and Savannakhet Province has an increased risk of HIV-infected patients. The development of a specific medical care system to combat helminth infection in HIV-infected patients is necessary.
